# A web-based radiomics nomogram combining MRI features and clinical data for predicting 30-day progression of acute ischemic stroke: a two-center study

**DOI:** 10.3389/fneur.2026.1774830

**Published:** 2026-08-10

**Authors:** Xiaohua Liu, Pugang Li, Huashuo Zhao, Wanruo Zhang, Hong Ma, Shaodong Li, Kai Xu, Liguo Dong

**Affiliations:** 1 Department of Radiology, The Affiliated Hospital of Xuzhou Medical University, Xuzhou, Jiangsu, China; 2 First School of Clinical Medicine, Xuzhou Medical University, Xuzhou, Jiangsu, China; 3 Department of Neurology, The 334 Affiliated Hospital of Nanchang University, Nanchang, Jiangxi, China; 4 School of Public Health, Xuzhou Medical University, Xuzhou, Jiangsu, China; 5 Department of Neurology, The Affiliated Hospital of Xuzhou Medical University, Xuzhou, Jiangsu, China

**Keywords:** AIS, dynamic nomogram, MRI, PIS, prediction model, radiomics

## Abstract

**Objective:**

To develop and validate an integrated nomogram combining MRI radiomics features with clinical variables for predicting 30-day progression risk in patients with acute ischemic stroke (AIS), and to deploy a web-based visualization tool for clinical application.

**Methods:**

This retrospective two-center study included 254 AIS patients. Radiomics features were extracted from DWI using MaZda, and key features were selected by LASSO logistic regression to build a radiomics signature. Clinical predictors were identified using logistic regression. Radiomics, clinical, and combined models were developed and evaluated using area under the curve (AUC), net reclassification index (NRI), and integrated discrimination improvement (IDI), calibration analysis, and decision curve analysis (DCA). Optimal cutoffs were determined in the training cohort using the Youden index and then directly applied to the external validation cohort without re-optimization. The corresponding training-derived thresholds were −1.2023 for the clinical model, −1.0368 for the radiomics model, and −2.3639 for the combined model. Bootstrap resampling (1,000 repetitions) was used for internal validation of the combined model.

**Results:**

Eight optimal radiomics features were selected from 300 extracted features. Multivariate analysis identified four independent predictors: Radiomics score (Radscore) (OR = 2.23, 95% CI: 1.28–5.11), National Institutes of Health Stroke Scale (NIHSS) score (OR = 1.57, 95% CI: 1.25–2.14), type 2 diabetes mellitus (OR = 9.28, 95% CI: 2.02–56.03), and monocytes (MO) (OR = 1.08, 95% CI: 1.02–1.16). The combined model showed favorable discriminative performance with AUCs of 0.945 (95%CI: 0.903–0.987) and 0.904 (95%*CI:* 0.854–0.954) in the training and validation cohorts, respectively. When the training-derived cutoff of −2.3639 was applied to the validation cohort, the combined model achieved 93.75% sensitivity, 73.95% specificity, and 83.85% balanced accuracy. Bootstrap internal validation yielded an optimism-corrected C-index of 0.912 and a corrected calibration slope of 0.769. The combined model outperformed the clinical-only and radiomics-only models, although the external validation should still be interpreted as an initial cross-center assessment.

**Conclusion:**

The integrated radiomics-clinical nomogram incorporating Radscore, NIHSS score, type 2 diabetes mellitus (T2DM), and MO showed potential for predicting 30-day progression risk in patients with AIS. This web-based tool may support early risk stratification, but further large-scale prospective validation is required before routine clinical application.

## Introduction

In China, cerebrovascular diseases have become the leading cause of mortality and a major contributor to disability, with approximately 80% of cases being ischemic strokes. The incidence of these strokes is increasing annually, and the age of onset is progressively decreasing. Data indicate that progressive ischemic stroke (PIS) accounts for 26.4–34% of cases among patients with acute ischemic stroke (AIS). If stroke progression is not promptly controlled, it can lead to higher mortality and disability rates, imposing a heavy burden on families and society (1, 2). Early identification of PIS is crucial for guiding clinical treatment decisions and facilitating timely neurological recovery.

Magnetic resonance imaging (MRI), with its excellent tissue resolution, serves as a key diagnostic tool for early detection. It allows direct and accurate observation of brain tissue changes at different time points after ischemia through tissue windows, enabling assessment of damage extent. This facilitates rapid definitive diagnosis and secures optimal treatment windows (3). In recent years, the rapid advancement of radiomics has opened new avenues for PIS prediction. MRI-based radiomics, independent of subjective radiologist evaluations, extracts high-dimensional quantitative features from complex medical images and converts them into high-throughput data. By mining these data, it uncovers pathophysiological information that is difficult to obtain through visual observation alone, earning it the moniker of “digital biopsy” in radiology. MRI radiomics has been applied to predict stroke recurrence (4, 5), hemorrhagic transformation (6), and outcomes of thrombectomy (7, 8). Existing predictive models primarily rely on clinical factors such as National Institutes of Health Stroke Scale (NIHSS) scores, blood pressure, blood glucose levels, intracranial artery stenosis, and inflammatory markers, but their predictive value remains limited (9, 10).

Early neurological progression after AIS remains clinically important because deterioration during the early post-stroke period may lead to prolonged hospitalization, increased monitoring needs, and worse subsequent recovery. Although the 90-day modified Rankin Scale (mRS) is widely used as a standard endpoint for long-term functional outcome assessment, 30-day endpoints have also been used as clinically meaningful short-term outcomes in stroke studies, including stroke or death, functional status, case fatality, and readmission (9–13). Therefore, identifying patients at high risk of 30-day progression may help optimize early monitoring and individualized management during the vulnerable early phase after AIS.

However, few studies have combined MRI-based radiomics features with independent clinical predictors to construct models for predicting 30-day progression in AIS patients, and most lack independent external validation. In this study, we aimed to develop and externally validate an integrated radiomics-clinical nomogram for predicting 30-day progression risk in patients with AIS.

## Materials and methods

### Study design

This retrospective, multicenter study was conducted in accordance with the Transparent Reporting of a Multivariable Prediction Model for Individual Prognosis or Diagnosis (TRIPOD) guidelines. The study protocol was approved by the institutional review boards of both participating centers.

### Ethics approval

This study was approved by the Institutional Review Boards of The Affiliated Hospital of Xuzhou Medical University (XYFY2021-KL107-01) and the 334th Hospital Affiliated to Nanchang University (2024-k006). Written informed consent was waived due to the retrospective nature of the study. All patient data were anonymized and handled in accordance with the Declaration of Helsinki and local data protection regulations.

### Patients

#### Patient selection

**Figure 1:**
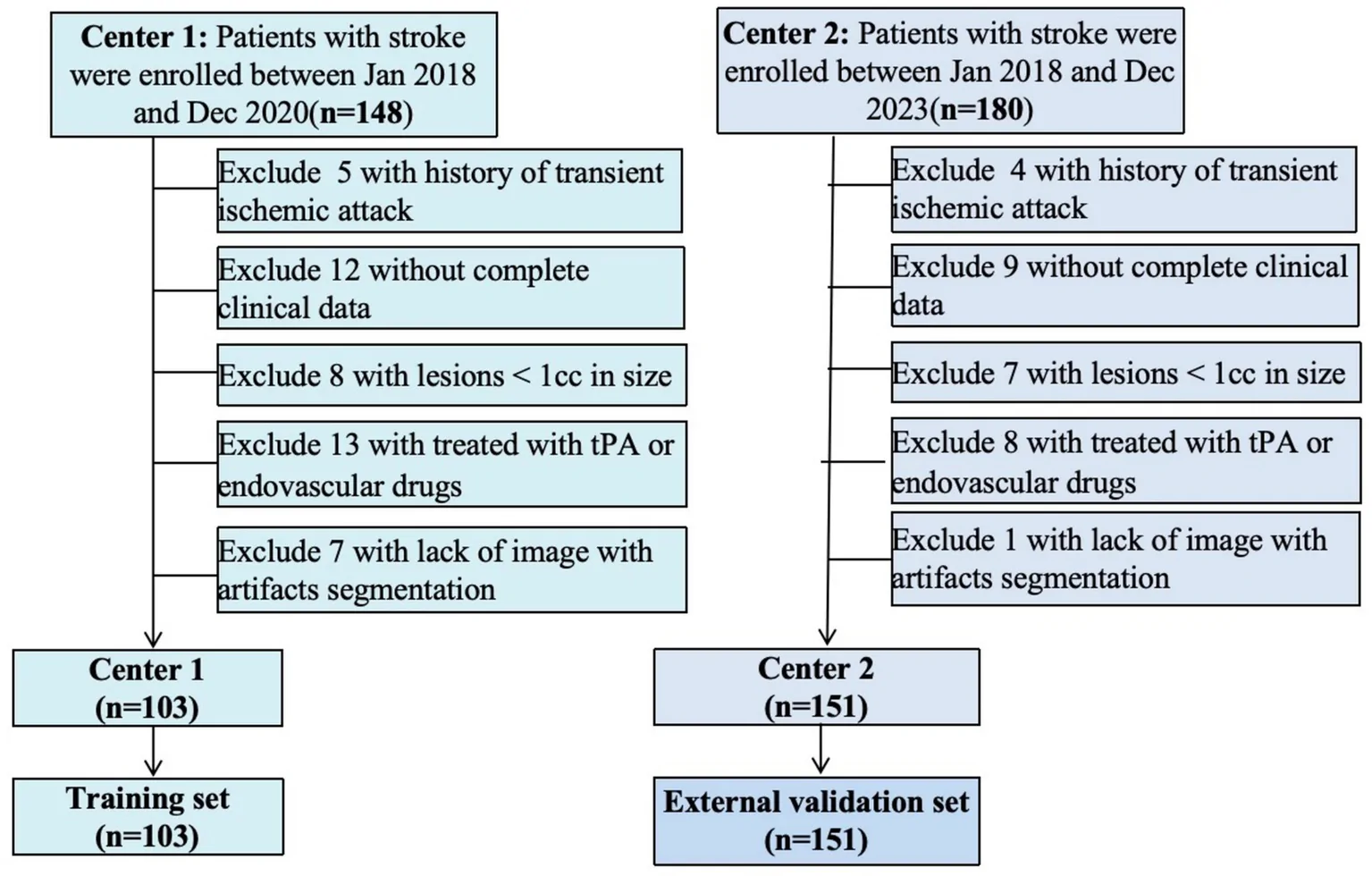
Flowchart of study patients and process.

Consecutive patients with acute ischemic stroke admitted within 24 h of symptom onset were retrospectively identified from two centers between August 2018 and December 2023. Inclusion criteria comprised: (1) First-ever AIS confirmed by neurologists according to the 2018 Chinese Guidelines (14); (2) DWI-MRI performed within 24 h of admission; (3) Age ≥18 years; (4) Complete clinical and imaging data available. Exclusion criteria included: (1) Transient ischemic attack; (2) Large-territory infarction (>1/3 middle cerebral artery territory); (3) Prior intravenous thrombolysis or endovascular treatment; (4) Severe comorbidities affecting prognosis; (5) Inadequate image quality for radiomics analysis. Patients receiving intravenous thrombolysis or endovascular therapy were excluded to reduce treatment-related confounding that could substantially influence early neurological progression, imaging manifestations, and associated clinical variables. In addition, because the overall rate of reperfusion therapy remained limited in China during the study period, this exclusion was considered acceptable for constructing a more homogeneous untreated cohort, although it also limits applicability to contemporary reperfusion-treated populations (15). Figure 1 provides a detailed flowchart of the patient selection process. In total, 254 patients were included in this study.

#### Clinical data

This retrospective study collected demographic and clinical variables recorded included age, sex, large artery atherosclerosis (LAA), NIHSS score at admission, pre-treatment levels of apolipoprotein B (APOB), lipoprotein(a) (LP(a)), homocysteine (HCY), low-density lipoprotein cholesterol (LDL-C), eosinophils (EO), basophils (BA), red blood cells (RBC), hemoglobin (HGB), mean platelet volume (MPV), platelet-large cell ratio (PLCR), lipoprotein-associated phospholipase A2 (Lp-PLA2), neutrophils (NE), white blood cells (WBC), monocytes (MO), platelets (PLT), total cholesterol (TC), triglycerides (TG), high-density lipoprotein cholesterol (HDL-C), lymphocytes (LY), plateletcrit (PCT), as well as history of essential hypertension (EH) and type 2 diabetes mellitus (T2DM). All patients were followed up at 7 days, 15 days, and 1 month after the AIS event via telephone interviews or MRI re-examinations. The primary endpoint was the occurrence of PIS progression within 30 days post-stroke, defined according to the diagnostic criteria from the European Progression of Stroke Study (16).

#### MRI acquisition and analysis

All patients underwent brain MRI using 3.0 T scanners (Discovery MR 750 W, GE Healthcare) with standardized protocols. DWI sequences were acquired with the following parameters: TR = 4,880 ms, TE = 82.7 ms, slice thickness = 6.0 mm, FOV = 24 × 24 cm, matrix = 160 × 160, b-value = 1,000 s/mm^2^.

#### Sample size calculation

The sample size was determined using a pragmatic power analysis to estimate the minimum number of subjects required to detect a clinically meaningful difference in the area under the curve (AUC) between diagnostic tests. Based on an expected AUC of 0.7 for the primary diagnostic test versus 0.9 for the secondary test (difference of 0.2), with 80% statistical power and alpha = 0.05, a total of 100 subjects were required (20 subjects in the positive group and 80 subjects in the negative group). Sample size calculations were performed using PASS software (version 25.0.2, NCSS, LLC, Kaysville, UT, USA). Given the high-dimensional nature of radiomics, this calculation should be interpreted as an exploratory reference rather than a formal justification that fully eliminates concerns regarding model optimism or overfitting.

#### Study design and data sources

This study employed a two-center design with temporal external validation. The training cohort was sourced from Center 1, while the external validation cohort was obtained from Center 2. To assess potential inter-center heterogeneity, baseline characteristics between the training cohort and the external validation cohort were formally compared. This design allowed an initial cross-center evaluation of model performance and helped assess potential overfitting from the training phase.

#### Radiomics feature extraction and selection

**Figure 2:**
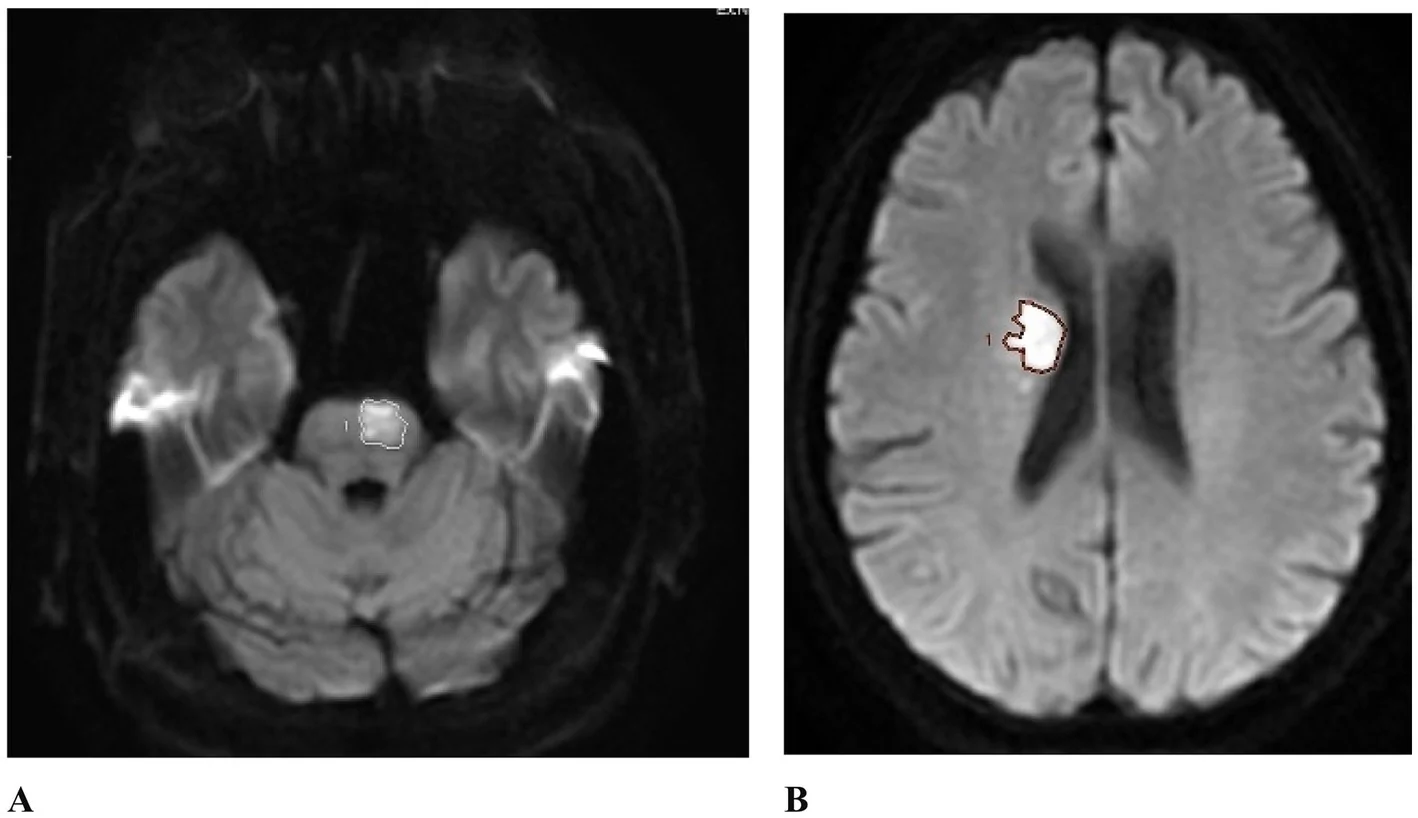
Lesion ROI delineation **(A)** NonPIS, **(B)** PIS.

The radiomics analysis followed the Image Biomarker Standardization Initiative (IBSI) guidelines. All data consisted of b = 1,000 DWI sequences stored in DICOM format with a resolution of 160×160. Radiomics analysis of the acute infarct regions in AIS patients was performed using MaZda 4.6 software. For the primary segmentation, the region of interest (ROI) was manually delineated along the lesion boundary on the largest axial slice by a radiologist with 10 years of experience in neuroimaging, as shown in Figure 2. In the present retrospective workflow, radiomics features were extracted from the original DWI images available in the clinical archive, and ADC maps were not included in the current analysis. More detailed voxel-level preprocessing parameters, such as standardized resampling and intensity normalization settings, were not prospectively predefined and could not be fully reconstructed; this should therefore be regarded as a limitation when interpreting reproducibility.

To evaluate the robustness of radiomics feature extraction against reader variation, 30 patients were randomly selected for reproducibility analysis. ROI delineation was independently performed by two radiologists with 8 years of experience in neuroimaging. Intraclass correlation coefficients (ICCs) were calculated to assess inter-observer agreement of the extracted radiomics features. Features with ICC > 0.75 were considered reproducible and retained for subsequent analysis.

A total of 300 quantitative features were extracted from the DWI images, categorized into seven groups: gray-level histogram (Histogram), absolute gradient (GRA), gray-level co-occurrence matrix (GLCM), gray-level run-length matrix (GLRLM), autoregressive model (ARM), wavelet transform (WAV), and gray-level size zone matrix (GLSZM). Because DWI is highly sensitive to acute ischemic lesions and was consistently available for all enrolled patients, only DWI-based radiomics features were analyzed in the present study. This single-sequence design may limit the amount of complementary imaging information and has therefore been acknowledged as a limitation.

To reduce feature redundancy, least absolute shrinkage and selection operator (LASSO) logistic regression with ten-fold cross-validation was performed in the training cohort. Features with non-zero coefficients were retained, and the radiomics signature (Radscore) was calculated as a linear combination of the selected features weighted by their corresponding coefficients.

#### Clinical variable selection

Variables demonstrating potential predictive value (*p* < 0.05) from univariate analysis were incorporated into stepwise multivariate logistic regression. Variable selection employed *p* < 0.05 for entry and *p* > 0.10 for removal, combined with the Akaike Information Criterion (AIC) as the stopping rule to optimize model parsimony and prevent overfitting.

#### Model development and construction

Three predictive models were developed and systematically compared: (1) Radiomics-only model based solely on the radiomics score (Radscore) derived from *LASSO* regression; (2) Clinical-only model incorporating only clinical and laboratory variables; (3) Combined model integrating both radiomics features and clinical variables.

### Model evaluation and validation

#### Training cohort

Discrimination performance was assessed through Receiver operating characteristic (ROC) analysis with AUC calculation and 95% confidence intervals using the pROC package. Sensitivity, specificity, accuracy, and balanced accuracy were calculated at the optimal cutoff. For each model, the optimal threshold was determined in the training cohort by maximizing the Youden index.

Calibration assessment included *Hosmer-Lemeshow* goodness-of-fit test (*p* > 0.05 indicating good acceptable calibration), calibration plots generated via bootstrapping with 1,000 resamples, and bootstrap-based estimation of optimism-corrected performance for the combined model, including the corrected C-index and calibration slope.

Clinical utility evaluation was performed using decision curve analysis (DCA) to evaluate the net benefit of the model across different threshold probabilities. Net benefit calculation was compared to treat-all and treat-none strategies. The clinical impact curve was used to assess the number of high-risk patients identified.

#### External validation cohort

The predictive models developed in the training cohort were applied to the independent validation cohort to assess generalizability. Discriminative performance was assessed through ROC curve analysis and AUC calculation with 95% confidence intervals. Sensitivity, specificity, accuracy, and balanced accuracy were calculated using the same fixed cutoff values derived from the training cohort, without re-estimating the threshold in the validation cohort. The fixed thresholds applied to the validation cohort were −1.2023 for the clinical model, −1.0368 for the radiomics model, and −2.3639 for the combined model.

Calibration performance was evaluated using calibration curves to assess agreement between predicted and observed probabilities, Hosmer-Lemeshow test for goodness-of-fit assessment, and calibration slope and intercept evaluation.

### Model comparison and selection

Net Reclassification Improvement (NRI) and Integrated Discrimination Improvement (IDI) analyses were performed to compare the performance of the three models (radiomics-only, clinical-only, and combined). These metrics provide complementary information to traditional discrimination measures and assess the clinical value of adding new predictors to existing models.

### Web-based nomogram development

Based on the optimal predictive model, a dynamic web-based nomogram was developed using appropriate software frameworks to facilitate clinical application and provide real-time risk assessment for individual patients. The nomogram interface was designed to be user-friendly for clinical implementation.

### Statistical analysis

All statistical analyses were performed using R software (version 4.3.2). Continuous variables were expressed as mean ± standard deviation or median (interquartile range) according to distribution normality assessed by the *Shapiro–Wilk* test. Categorical variables were presented as frequencies and percentages. Between-group comparisons were performed using Student’s *t*-test or *Mann–Whitney* U test for continuous variables and Chi-square test or *Fisher’s* exact test for categorical variables. In addition, baseline characteristics between the training cohort and the external validation cohort were formally compared to evaluate cohort comparability and potential inter-center heterogeneity. Statistical significance was set at *p* < 0.05, and all tests were two-sided.

## Results

### Patient characteristics

A total of 254 patients was included in the final analysis, with 54 (21.3%) developing PIS within 30 days. The training cohort comprised 103 patients (22 with PIS, 21.4%), while the validation cohort included 151 patients (32 with PIS, 21.2%).

Baseline characteristics of the training cohort according to PIS status are summarized in Table 1. The comparison of baseline characteristics between the training cohort and the external validation cohort is provided in Supplementary Table S1. In the training cohort, there were no statistically significant differences between the PIS and non-PIS groups in terms of age, gender, HCY, LDL-C, HDL-C, TC, LY, EO, BA, RBC, HGB, PCT, MPV, and PLCR (all *p* > 0.05). However, significant differences were observed between the PIS and non-PIS groups in LAA, NIHSS score, EH, T2DM, TG, APOB, WBC, NE, MO, PLT, and Lp-PLA2 (all *p* < 0.05).

**Table 1:** Baseline data of clinic characteristics of patients with nonPIS in PIS group.

Variables	Training cohort (*n* = 103)			External validation cohort (*n* = 151)		
	nonPIS group (*n* = 81)	PIS group (*n* = 22)	*p* value	nonPIS group (*n* = 119)	PIS group (*n* = 32)	*p* value
Age (Yrs)	62.84 ± 9.08	65.27 ± 8.81	0.262	72.69 ± 11.12	68.66 ± 13.50	0.128
Gender			0.121			0.022
Male	55 (67.90%)	11 (50.00%)		75 (63.03%)	27 (84.38%)	
Female	26 (32.10%)	11 (50.00%)		44 (36.97%)	5 (15.62%)	
LAA			0.002			0.572
No	37 (45.68%)	2 (9.09%)		12 (10.08%)	5 (15.62%)	
Yes	44 (54.32%)	20 (90.91%)		107 (89.92%)	27 (84.38%)	
NIHSS	4.00 [3.00;5.00]	7.00 [7.00;14.00]	<0.0001	3.00 [2.00;4.50]	6.00 [4.00;8.00]	<0.0001
EH			0.011			<0.0001
No	39 (48.15%)	4 (18.18%)		66 (55.46%)	6 (18.75%)	
Yes	42 (51.85%)	18 (81.82%)		53 (44.54%)	26 (81.25%)	
T2DM			0.007			0.001
No	64 (79.01%)	11 (50.00%)		83 (69.75%)	12 (37.50%)	
Yes	17 (20.99%)	11 (50.00%)		36 (30.25%)	20 (62.50%)	
HCY (umol/L)	13.55 [10.50;16.52]	13.46 [11.20;16.96]	0.673	11.40 [8.34;15.62]	12.75 [8.55;17.61]	0.334
LDL-C (mmol/L)	2.78 ± 1.04	3.12 ± 0.98	0.159	2.55 ± 0.91	2.37 ± 0.65	0.216
HDL-C (mmol/L)	1.10 [0.93;1.29]	1.06 [0.89;1.19]	0.355	1.40 [1.15;1.69]	1.50 [1.20;1.83]	0.290
TG (mmol/L)	1.32 [0.97;1.74]	1.50 [1.26;2.35]	0.032	1.27 [0.76;1.99]	1.61 [1.23;2.10]	0.023
APOB (g/L)	0.88 ± 0.27	1.02 ± 0.25	0.032	0.89 ± 0.26	1.03 ± 0.19	0.001
TC (mmol/L)	4.46 [3.77;5.33]	4.82 [4.40;5.28]	0.212	4.53 [3.96;5.40]	4.68 [3.82;5.28]	0.918
WBC (10^9/L)	6.00 [5.30;7.00]	7.15 [6.20;8.70]	0.003	6.69 [5.58;7.62]	7.58 [6.97;8.89]	0.001
NE (10^9/L)	3.95 [3.39;4.64]	5.11 [4.30;6.22]	<0.0001	4.31 [3.38;5.54]	5.21 [4.40;6.58]	0.004
LY (10^9/L)	1.50 [1.30;1.80]	1.35 [1.00;1.80]	0.150	1.50 [1.12;1.90]	1.55 [1.06;2.08]	0.929
MO (10^9/L)	0.33 [0.27;0.40]	0.42 [0.31;0.46]	0.004	0.41 [0.31;0.50]	0.41 [0.34;0.46]	0.859
EO (10^9/L)	0.09 [0.05;0.15]	0.09 [0.04;0.15]	0.981	0.10 [0.05;0.19]	0.10 [0.04;0.20]	0.746
BA (10^9/L)	0.01 [0.00;0.02]	0.01 [0.01;0.02]	0.814	0.03 [0.02;0.04]	0.03 [0.02;0.05]	0.780
RBC (10^12/L)	4.64 [4.22;4.86]	4.55 [4.28;4.95]	0.714	4.27 [3.92;4.71]	4.41 [4.00;4.76]	0.539
HGB (g/L)	141.00 [130.00;149.00]	137.00 [132.00;149.00]	0.714	131.00 [119.50;145.50]	137.00 [125.25;151.00]	0.197
PLT (10^9/L)	200.00 [174.00;232.00]	238.00 [191.00;276.00]	<0.0001	194.00 [142.50;233.50]	237.00 [206.00;285.50]	<0.0001
lp-pla2 (ng/mL)	164.00 [110.00;332.00]	436.00 [327.00;487.00]	<0.0001	184.00 [131.00;395.50]	421.00 [306.50;487.00]	<0.0001
PCT (%)	0.22 [0.18;0.25]	0.26 [0.19;0.31]	0.057	0.22 [0.17;0.26]	0.20 [0.16;0.24]	0.231
MPV (fl)	10.71 ± 1.51	10.20 ± 1.06	0.075	10.15 ± 0.91	10.17 ± 0.90	0.905
PLCR	29.40 [24.10;38.00]	27.45 [21.60;31.50]	0.125	24.90 [20.00;30.40]	26.15 [20.00;31.05]	0.825

A formal comparison of baseline characteristics between the training cohort and the external validation cohort was additionally performed. Significant differences were observed in age, LAA, NIHSS, HCY, LDL-C, HDL-C, WBC, MO, BA, RBC, HGB, MPV, and PLCR, whereas sex, EH, T2DM, TG, APOB, TC, NE, LY, EO, PLT, lp-pla2, and PCT did not differ significantly between the two cohorts (Supplementary Table S1). These findings indicate that the two cohorts were not fully comparable and that inter-center heterogeneity was present.

### Radiomics feature extraction and radiomics model construction

**Figure 3:**
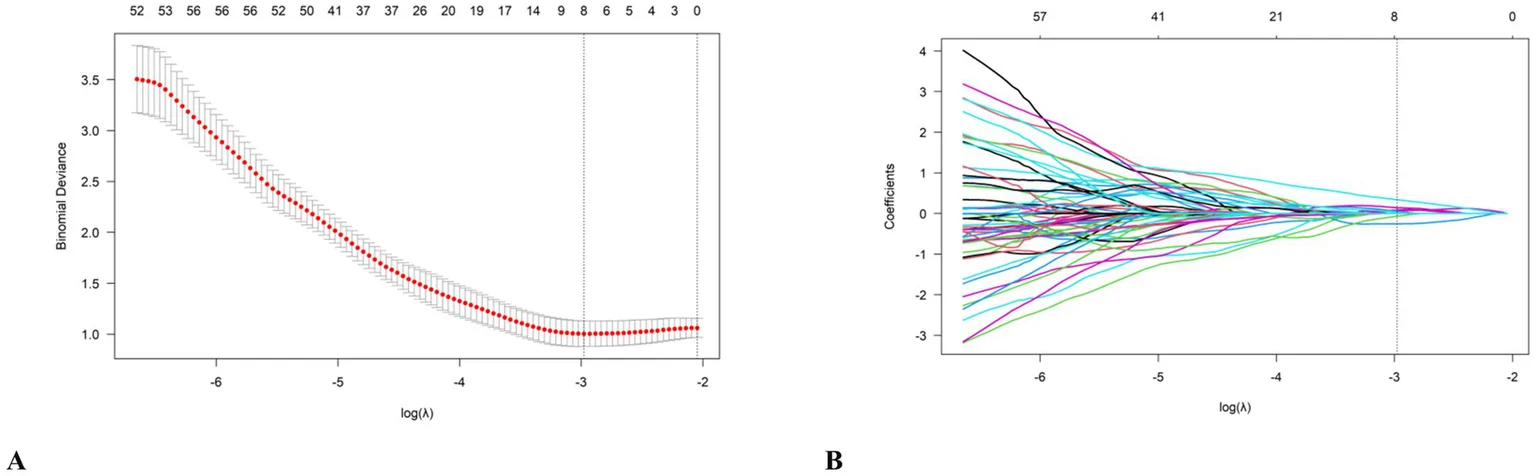
Selection of optimal parameters for the *LASSO*-*Logistic* Regression Model based on 10-fold cross-validation. **(A)** The vertical dashed line indicates the log(*λ*) value corresponding to the optimal λ, with the selection criterion based on minimizing the deviance. A total of 8 features were selected in this study. **(B)** Illustrates the coefficient convergence plot for feature selection using 10-fold cross-validation in the *LASSO* model. Each curve in the figure represents the trajectory of coefficient changes for an individual independent variable. The vertical line corresponds to the non-zero features identified after 10-fold cross-validation, resulting in a total of 8 selected features.

From the initial 300 radiomics features extracted from DWI images, LASSO-logistic regression with 10-fold cross-validation identified 8 optimal features with non-zero coefficients (Figures 3A,B). The selected features included: Feature 1: _Area_S(0,1); Feature 2: S(1,-1)SumOfSqs; Feature 3: _Area_S(0,2); Feature 4: S(3,-3)Correlat; Feature 5: S(3,-3)InvDfMom; Feature 6: S(0,4)Correlat; Feature 7: Horzl_LngREmph; Feature 8: WavEnLH_s-4 (Supplementary Table S2).

Based on these 8 features and their corresponding coefficient weights, the radiomics signature was constructed, and the radiomics score (Radscore) model was formulated as follows: Radscore = −1.434 + 0.065 × Feature 1–0.253 × Feature 2 + 0.146 × Feature 3 + 0.341 × Feature 4 + 0.085 × Feature 5 + 0.055 × Feature 6 + 0.029 × Feature 7–0.067 × Feature 8.

### Model development and evaluation

#### Model construction methodology

A comprehensive predictive model for AIS progression was developed using the training cohort data. Univariate and stepwise multivariate logistic regression analyses were performed on clinical and radiomics features from 103 AIS patients. The detailed results are presented in Table 2.

**Table 2:** Univariate and multivariate l*ogistic r*egression analysis for clinical data selection in model development.

Variable	Univariate analysis		Multivariate analysis	
	OR (95%*CI*)	*p* value	OR (95%*CI*)	*p* value
Rad.score	2.72 (1.71–4.96)	<0.0001	2.23 (1.28–5.11)	0.023
LAA (Yes)	8.41 (2.25–54.87)	0.006		
NIHSS	1.50 (1.27–1.84)	<0.0001	1.57 (1.25–2.14)	0.001
EH (Yes)	4.18 (1.41–15.44)	0.016		
T2DM (Yes)	3.76 (1.40–10.32)	0.009	9.28 (2.02–56.03)	0.007
TG	1.14 (0.78–1.62)	0.435		
APOB	6.41 (1.11–41.21)	0.042		
WBC	1.51 (1.15–2.03)	0.004		
NE	1.65 (1.23–2.28)	0.001		
MO	1.06 (1.02–1.11)	0.005	1.08 (1.02–1.16)	0.019
PLT	1.01 (1.00–1.02)	0.060		
lppla2	1.01 (1.00–1.01)	<0.0001		
TOAST	0.36 (0.14–0.69)	0.008		

Univariate *logistic* regression analysis identified 11 parameters, significantly associated with AIS progression (*p* < 0.05): Radscore, LAA, NIHSS score, EH, T2DM, APOB, WBC, NE, MO, Lp-PLA2, and TOAST classification. Stepwise multivariate *logistic* regression analysis identified Radscore (OR = 2.23, 95% *CI*: 1.28–5.11, *p* = 0.023), NIHSS (OR = 1.57, 95% *CI*: 1.25–2.14, *p* = 0.001), T2DM (OR = 9.28, 95% *CI*: 2.02–56.03, *p* = 0.007), and MO (OR = 1.08, 95% *CI*: 1.02–1.16, *p* = 0.019) as statistically significant predictors of AIS progression (all *p* < 0.05), serving as independent risk factors for the predictive model (OR > 1).

#### Construction of three predictive models

To comprehensively evaluate model performance, three distinct models were constructed based on the multivariate logistic regression results: Clinical model incorporating NIHSS score, T2DM, and MO as clinical risk factors. Radiomics model based solely on the Radscore, and Combined model integrating both clinical factors and radiomics features.

#### Model performance assessment and comparison

##### Discriminative performance analysis

**Figure 4:**
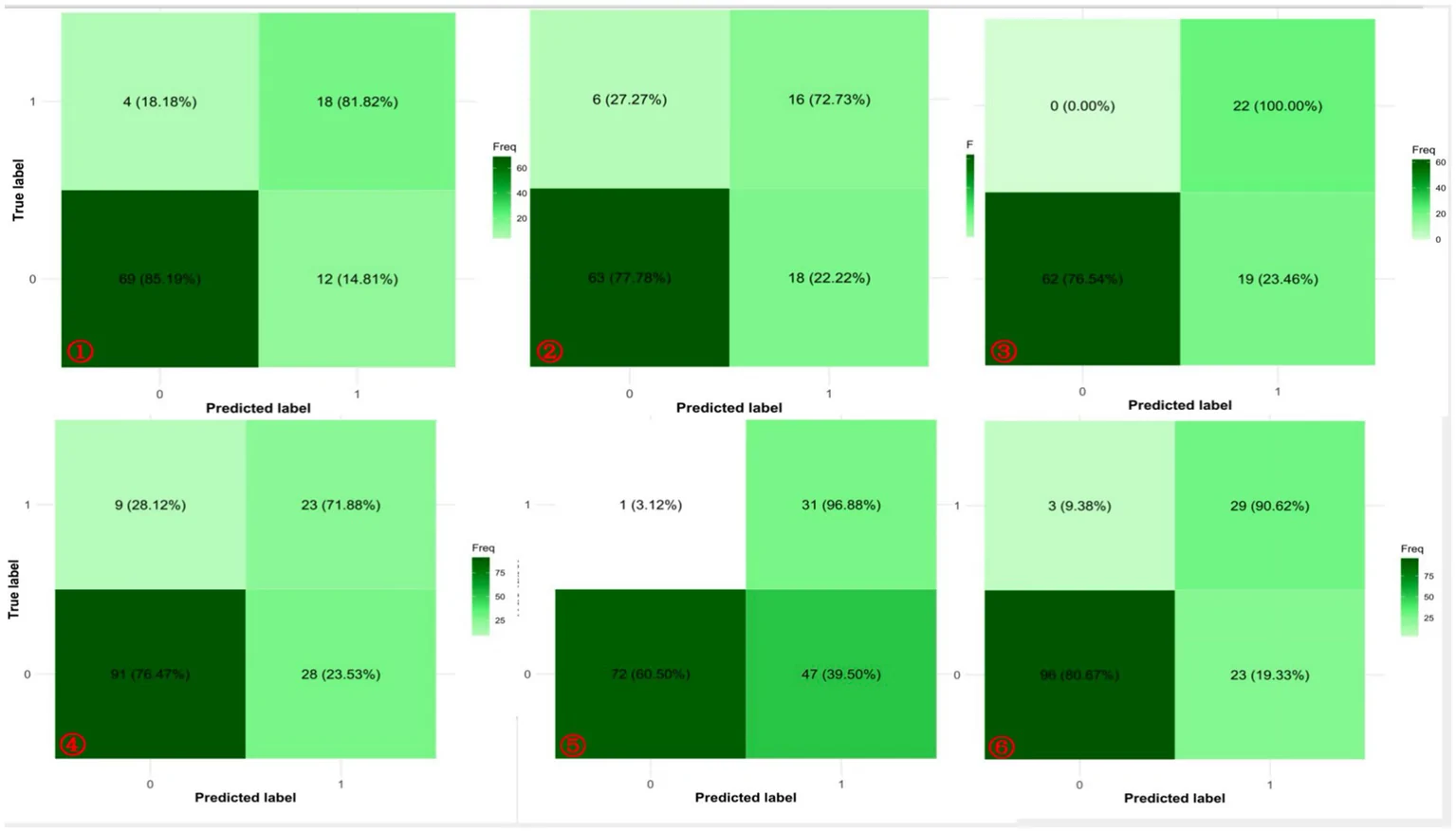
Confusion matrix (training cohort: 1 = Clinical model, 2 = Radiomics model, 3 = Combined model; validation cohort: 4 = Clinical model, 5 = Radiomics model, 6 = Combined model).

**Figure 5:**
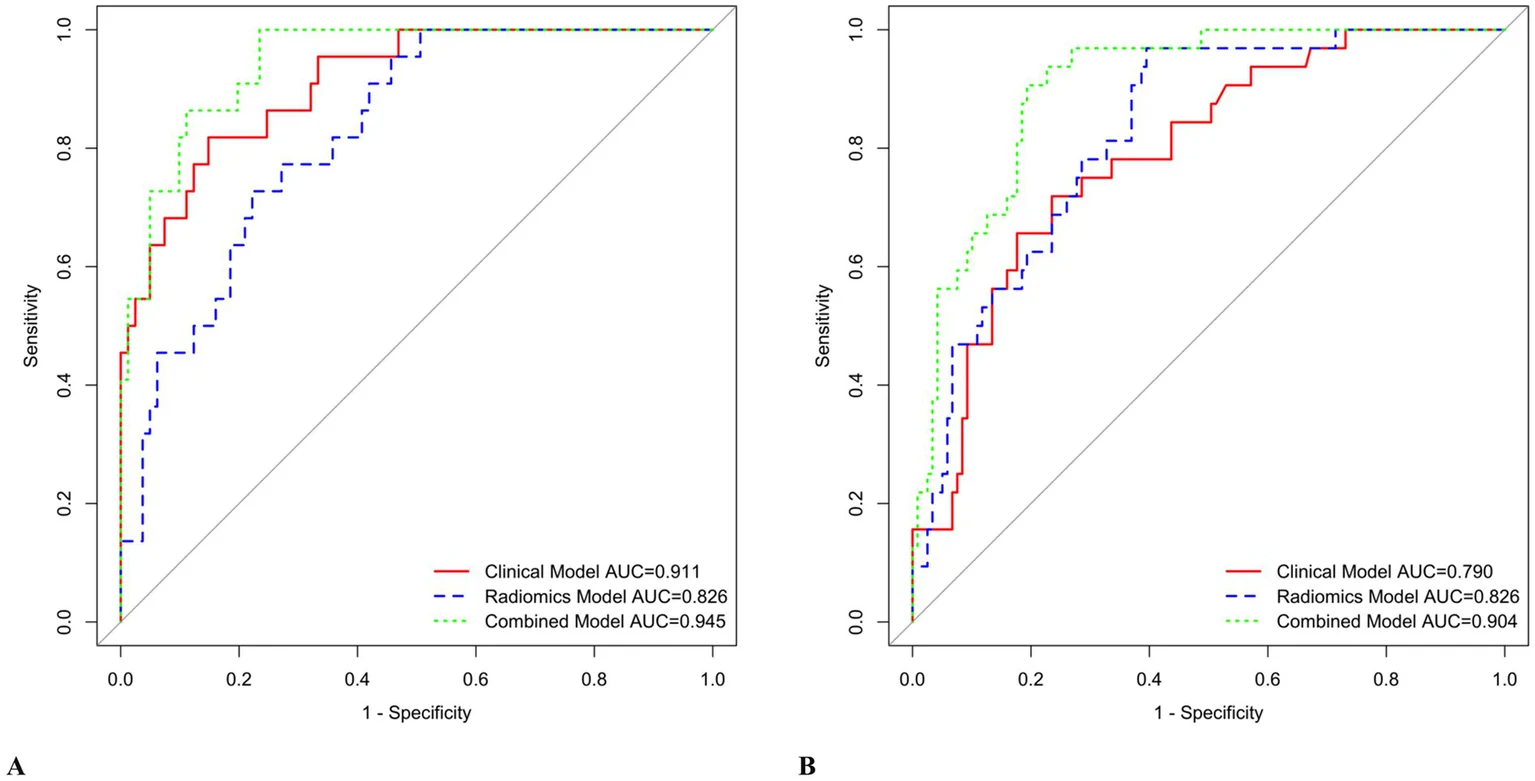
ROC curves of PIS. **(A)** ROC curves in the training cohort. **(B)** ROC curves in the validation cohort.

ROC curve analysis (Table 3; Figures 4, 5) demonstrated that the combined model achieved superior discriminative performance in both training and validation cohorts. In the training cohort, the combined model achieved an AUC of 0.945 (95% *CI*: 0.903–0.987), significantly outperforming the clinical model (AUC = 0.911, 95% *CI*: 0.847–0.974) and radiomics model (AUC = 0.826, 95% *CI*: 0.740–0.912). In the external validation cohort, the combined model maintained superior performance with an AUC of 0.904 (95% *CI*: 0.854–0.954), compared to the clinical model (AUC = 0.790, 95% *CI*: 0.706–0.874) and radiomics model (AUC = 0.826, 95% *CI*: 0.754–0.897). When the fixed training-derived cutoffs of −1.2023, −1.0368, and −2.3639 were applied to the clinical, radiomics, and combined models, respectively, the combined model showed an accuracy of 0.7815, sensitivity of 0.9375, specificity of 0.7395, and balanced accuracy of 0.8385. The corresponding values were 0.7815, 0.6562, 0.8151, and 0.7357 for the clinical model, and 0.7020, 0.7812, 0.6807, and 0.7310 for the radiomics model.

**Table 3:** Comparison of predictive and evaluation metrics for three models in training and external validation cohorts.

Models	AUC (95%CI)	Accuracy (95%CI)	Sensitivity	Specificity
Training cohort				
Clinical model	0.911 (0.847–0.974)	0.845 (0.760–0.908)	0.818	0.852
Radiomics model	0.826 (0.740–0.912)	0.767 (0.673–0.844)	0.727	0.778
Combined model	0.945 (0.903–0.987)	0.816 (0.727–0.885)	1.000	0.765
External validation cohort				
Clinical model	0.790 (0.706–0.874)	0.782 (0.707–0.845)	0.656	0.815
Radiomics model	0.826 (0.754–0.897)	0.702 (0.622–0.774)	0.781	0.681
Combined model	0.904 (0.854–0.954)	0.782 (0.707–0.845)	0.938	0.740

##### Model calibration assessment

**Figure 6:**
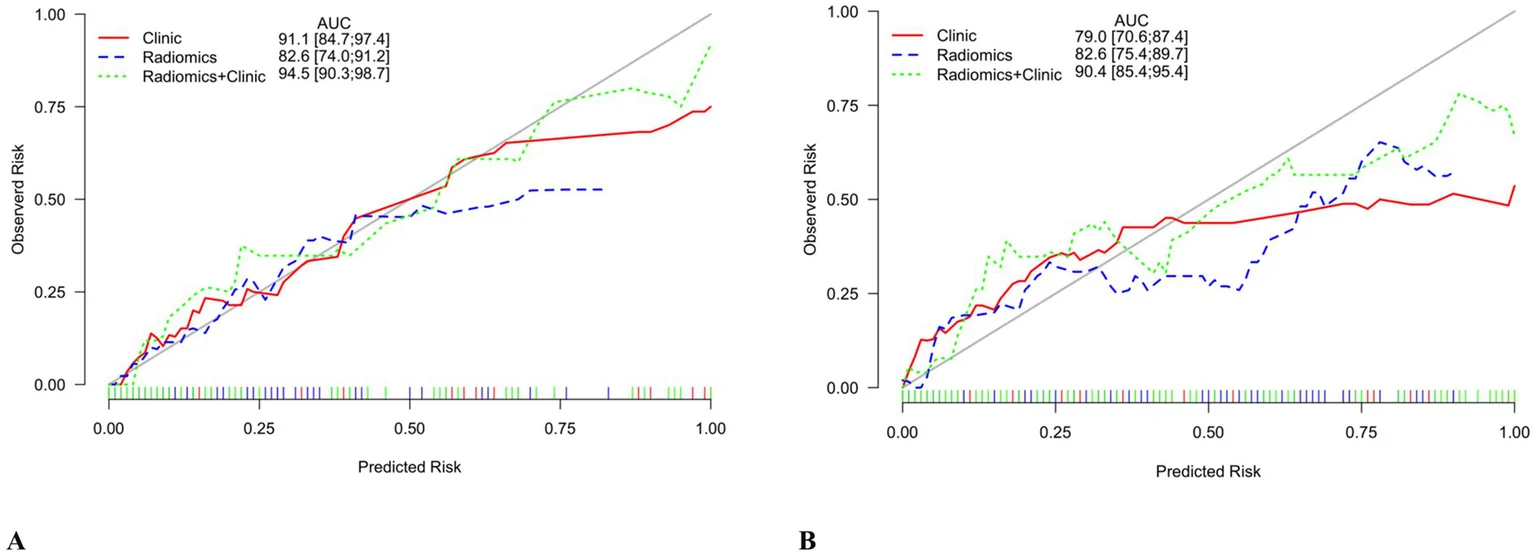
Calibration curves of PIS. **(A)** Calibration curves in the training cohort. **(B)** Calibration curves in the validation cohort.

Calibration curve analysis and *Hosmer-Lemeshow* goodness-of-fit tests (Figure 6) revealed acceptable calibration for all three models in the training cohort: clinical model (χ^2^ = 1.407, *p* = 0.843), radiomics model (χ^2^ = 3.649, *p* = 0.456), and combined model (χ^2^ = 2.451, *p* = 0.654). However, in the validation cohort, only the combined model demonstrated good calibration (χ^2^ = 5.076, *p* = 0.280), while both the clinical model (χ^2^ = 23.833, *p* = 0.0001) and radiomics model (χ^2^ = 14.588, *p* = 0.006) showed poor calibration. Bootstrap internal validation of the combined model yielded a corrected Dxy of 0.8244, corresponding to an optimism-corrected C-index of 0.9122, and a corrected calibration slope of 0.7686. These results suggest some degree of optimism, but the combined model retained good corrected discrimination and comparatively better calibration than the single-model approaches.

##### Model improvement metrics

NRI and IDI analyses demonstrated significant improvements of the combined model compared to the radiomics model: NRI = 1.192 (95% *CI*: 0.822–1.562, *p* < 0.0001); IDI = 0.305 (95% *CI*: 0.178–0.432, *p* < 0.0001). Similarly, the combined model showed significant improvement over the clinical model: NRI = 0.846 (95% *CI*: 0.460–1.233, *p* < 0.0001); IDI = 0.079 (95% *CI*: 0.026–0.131, *p* = 0.004).

##### Clinical utility assessment

**Figure 7:**
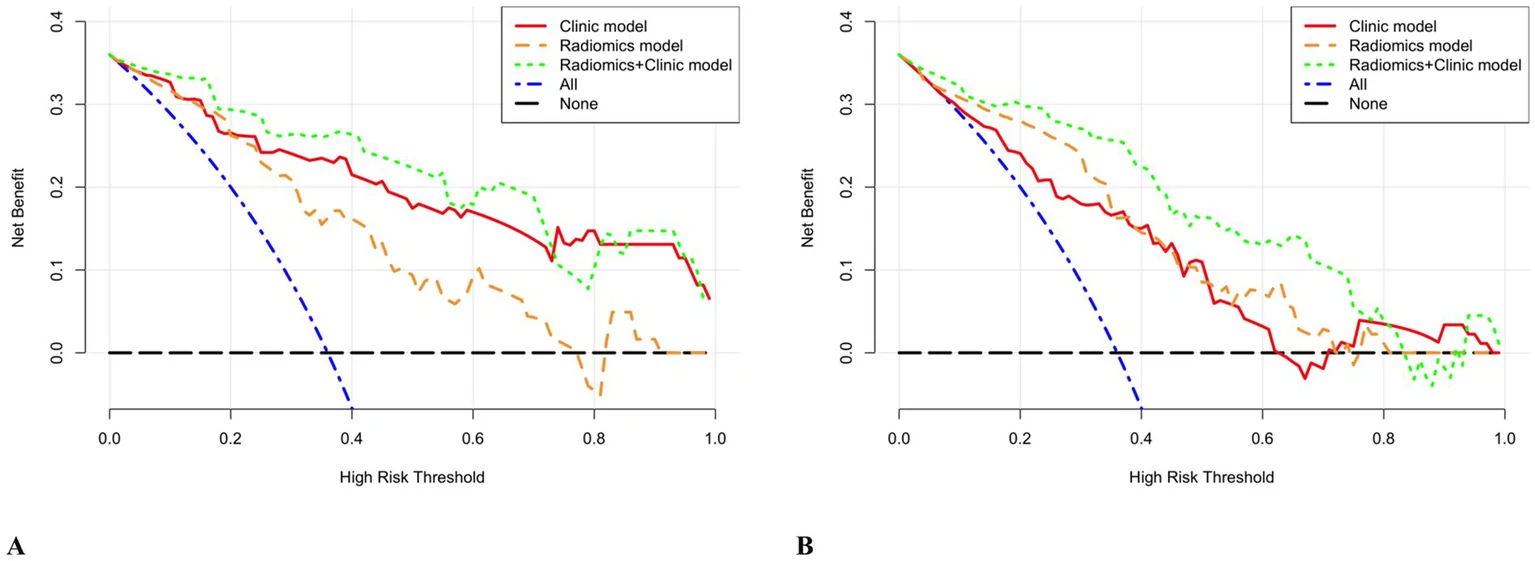
DCA curves of PIS. **(A)** DCA in the training cohort **(B)** DCA in the validation cohort. DCA, decision curve analysis.

DCA results (Figure 7) demonstrated that the combined model provided the highest net benefit across a wide range of threshold probabilities (0.1–0.8), surpassing other models and both “treat all” and “treat none” strategies, indicating superior clinical utility.

#### Nomogram construction

**Figure 8:**
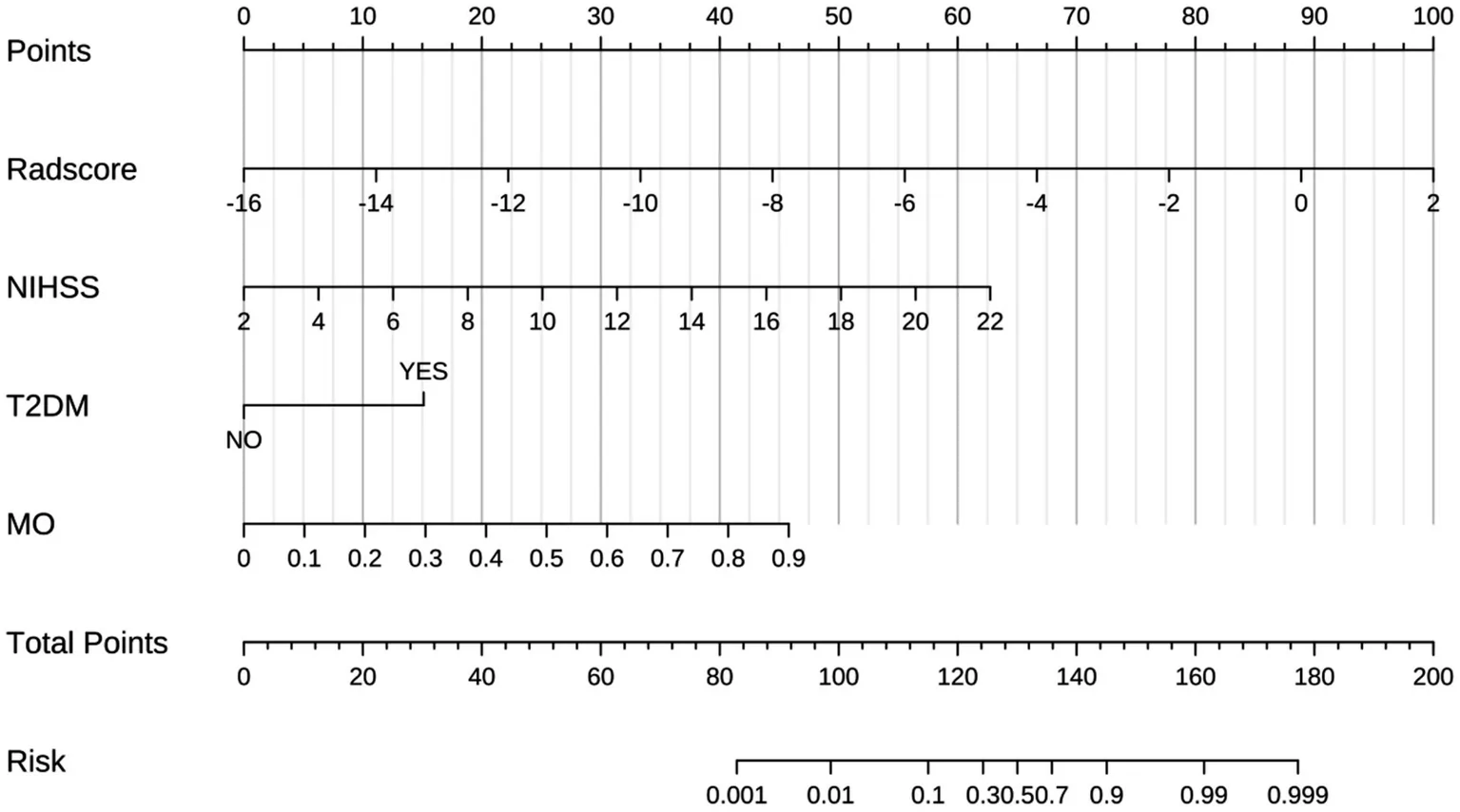
Nomogram for predicting the 30-day progression risk.

Based on the four identified predictive variables, a nomogram for the combined model was constructed using the ‘rms’ package in R software (Figure 8), providing an intuitive and convenient risk assessment tool for clinical application.

### Web-based nomogram deployment

**Figure 9:**
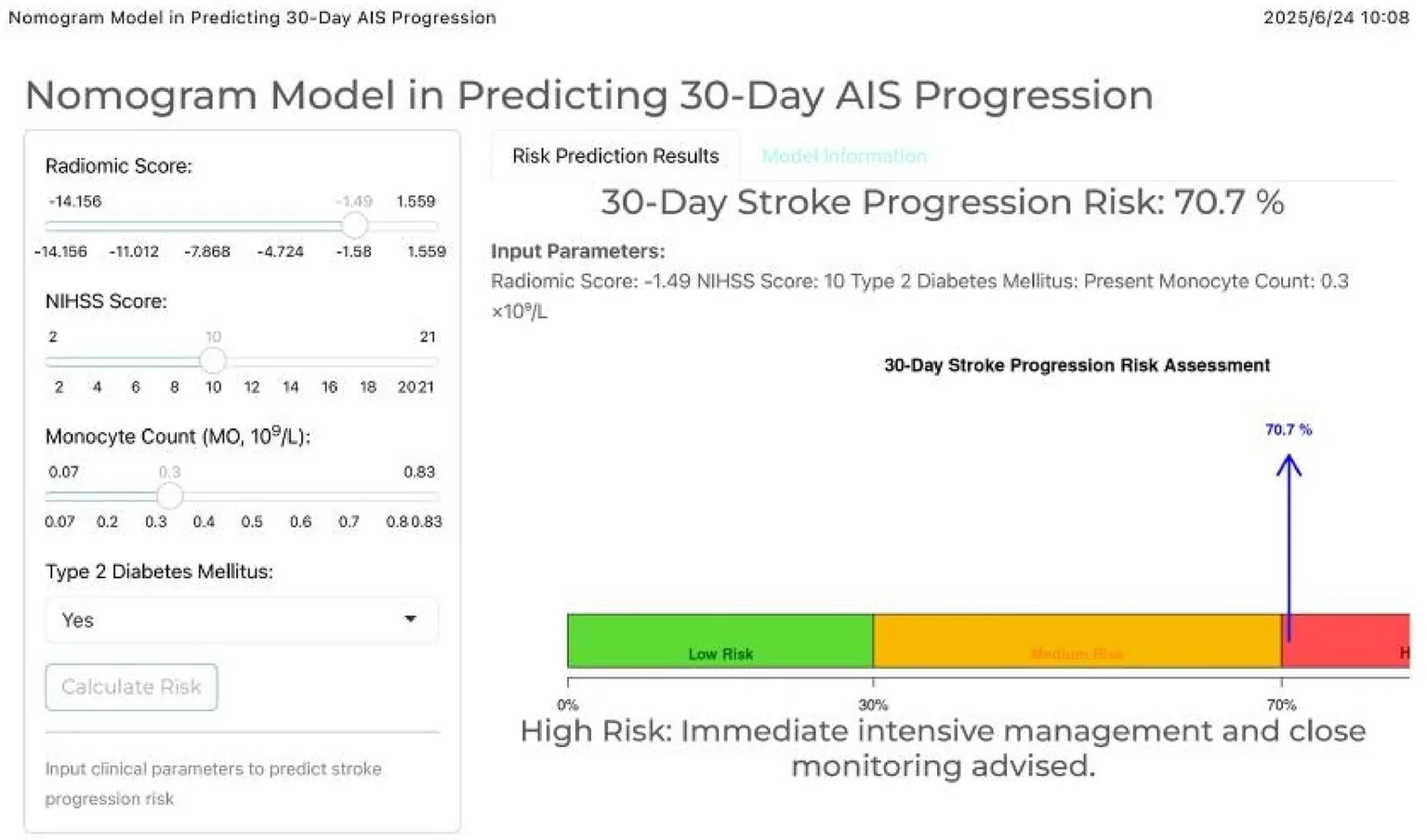
Operation page of web-based nomogram; PIS risk prediction at 30-day of the corresponding patient (blue line).

A dynamic, web-based nomogram was successfully developed and deployed (Figure 9). The tool allows clinicians to input patient-specific values for the four predictors and obtain real-time probability estimates for 30-day stroke progression. The nomogram is accessible at: https://myshinytest.shinyapps.io/DynNomapp/ and provides an intuitive interface for clinical decision-making.

## Discussion

This study developed and externally evaluated a web-based radiomics-clinical nomogram for predicting 30-day progression in patients with acute ischemic stroke. The combined model showed favorable discriminative performance and calibration in both the training and external validation cohorts and outperformed the radiomics-only and clinical-only models. Importantly, the combined model retained favorable performance in the external validation cohort even when the validation set was assessed using the fixed cutoffs derived from the training cohort rather than re-optimized thresholds. This stricter evaluation strategy reduces optimism related to threshold selection and provides a more conservative estimate of model generalizability. Because the present model was designed to predict early clinical deterioration within 30 days after AIS rather than long-term disability, these findings should be interpreted primarily as evidence for short-term risk stratification. The incremental value of radiomics should therefore be interpreted cautiously as a potentially useful adjunct to clinical assessment rather than definitive proof that radiomics alone provides unique biological information beyond conventional variables.

In the present study, we selected 30-day progression as the primary endpoint because our main objective was to predict early clinical deterioration rather than long-term disability. The early period after AIS represents a critical phase during which neurological worsening, recurrent ischemic events, and other complications may occur. A model that identifies patients at increased risk of short-term progression may support closer clinical surveillance, timely adjustment of treatment strategies, and more individualized allocation of medical resources. The clinical relevance of 30-day outcome assessment is also supported by previous stroke studies, which have used 30-day stroke or death, functional status, case fatality, and readmission as meaningful short-term endpoints (9–13). Nevertheless, although the 30-day endpoint is useful for early risk prediction, it should not be considered a substitute for long-term functional outcomes such as the 90-day mRS.

### Principal findings and clinical significance

PIS remains a critical challenge in contemporary stroke management, with its complex pathogenesis involving multifactorial etiologies driven by diverse pathogenic mechanisms (17). PIS profoundly impacts clinical outcomes and constitutes a leading cause of mortality and long-term disability in China (18). The accurate prediction of PIS risk, early identification of high-risk populations, and guidance for clinicians to monitor disease progression and implement timely interventions are crucial for preventing neurological deterioration and optimizing patient prognosis. However, individual risk factors demonstrate limited utility in identifying progressive stroke, with suboptimal predictive accuracy (19). Our study addresses this critical clinical need by developing a comprehensive, validated predictive model that integrates quantitative imaging biomarkers with established clinical risk factors.

The Rad-score emerged as an important predictor of PIS in the present study. Our analysis identified eight discriminative MRI radiomics features, comprising four co-occurrence matrix features, one run-length matrix feature, one wavelet transform feature, and two size-zone matrix features, with co-occurrence matrix features demonstrating the highest predictive weight. These quantitative texture features may capture lesion signal heterogeneity on DWI; however, because the selected MaZda-derived descriptors are not directly linked to specific histopathological substrates, their biological interpretation should be considered exploratory rather than definitive (20).

Any pathophysiological explanation of the selected radiomics features should be regarded as hypothesis-generating rather than directly demonstrated by our data. AIS progression is likely influenced by multiple interacting factors, including tissue vulnerability, hemodynamic status, occlusion characteristics, individual biological variation, and differential ischemic tolerance among neural and vascular cell populations (19, 21–26). These processes may contribute to imaging heterogeneity on DWI, but the present study was not designed to establish direct mechanistic links between individual texture features and specific biological pathways.

An important issue in the present study is the heterogeneity between the training cohort and the external validation cohort. Although the model was externally tested in an independent center, the two cohorts differed significantly in several demographic, clinical, and laboratory variables, including age, LAA, NIHSS, and multiple hematologic and lipid-related parameters. Such differences suggest that the patient composition was not fully comparable across centers and may have influenced model performance in the validation cohort. Therefore, the current external validation should be interpreted as an initial cross-center assessment rather than conclusive evidence of broad generalizability. Further validation in larger and more diverse external cohorts is still required.

### Comparison with existing literature and novel contributions

Our findings corroborate and extend previous research establishing type 2 diabetes mellitus (T2DM) as an independent risk factor for PIS occurrence (27). The pathophysiological mechanisms underlying this association involve a cascade of chronic hyperglycemia-induced vascular pathology, including endothelial dysfunction, accelerated atherosclerosis, and substantially elevated risk of ischemic events (27). Furthermore, metabolic disturbances associated with T2DM enhance platelet aggregation and promote prothrombotic states, while acute hyperglycemia exacerbates hypoxic–ischemic brain tissue damage and impairs neuronal energy metabolism and cellular repair processes (28). Multiple interconnected pathophysiological mechanisms contribute to adverse outcomes in diabetic stroke patients, including: elevated oxidative stress that compromises blood–brain barrier integrity and promotes cerebral edema formation (29); increased prevalence of comorbid cardiovascular diseases (30); upregulated matrix metalloproteinase activity leading to free radical accumulation and impaired clearance mechanisms, further accelerating atherosclerotic progression (31); and disrupted blood–brain barrier function with increased endothelial permeability that impedes effective collateral circulation establishment (32).

The incorporation of NIHSS score into our predictive framework aligns with extensive validation literature demonstrating its utility as a comprehensive, standardized assessment tool for neurological impairment in AIS patients (33). The NIHSS scores systematically evaluates multiple neurological domains, including level of consciousness, motor function, sensory function, and language abilities, providing a quantitative assessment of stroke severity and neurological deficit burden (33). Higher NIHSS scores demonstrate strong correlation with more severe neurological damage and poorer short- and long-term clinical outcomes (34, 35). Previous investigations have established NIHSS scores as a robust predictor of short-term mortality and functional outcomes, with predictive accuracy increasing over time post-stroke onset (34–36). Notably, community hospital-based studies have demonstrated that each 1-point increase in baseline NIHSS score confers a 2.3-fold increase in mortality risk and triples the likelihood of persistent ambulatory impairment (36). Our study further validates the critical importance of NIHSS scores as an essential component for predicting 30-day progression in AIS patients.

The role of monocytes in AIS pathophysiology represents an increasingly recognized area of clinical investigation. Following acute ischemic stroke onset, circulating monocytes rapidly infiltrate damaged brain parenchyma and undergo phenotypic differentiation into tissue macrophages, exerting complex dual roles in stroke pathophysiology (37, 38). During the acute phase, these inflammatory cells initially exacerbate neuroinflammation and secondary brain injury through release of pro-inflammatory mediators, reactive oxygen species, and matrix metalloproteinases that disrupt blood–brain barrier integrity (37, 39). Subsequently, during the subacute and chronic phases, differentiated macrophages contribute to tissue repair and neural regeneration processes by clearing cellular debris, phagocytosing apoptotic cells, and producing neurotrophic factors that support neuronal survival and axonal regeneration (37, 38). The monocyte-to-high-density lipoprotein cholesterol ratio (MHR) has emerged as a novel inflammatory biomarker with demonstrated predictive value for 30-day mortality in AIS patients (40), providing strong rationale for our inclusion of MO as a predictive variable in the integrated model.

### Clinical implications and translational impact

Our integrated nomogram model, combining NIHSS score, T2DM, MO, and quantitative radiomics features, showed improved predictive performance for 30-day disease progression in AIS patients compared with the single-model approaches. However, given the limited sample size, inter-center heterogeneity, and residual risk of optimism, these findings should be interpreted as preliminary evidence supporting the potential adjunctive value of radiomics rather than as definitive proof of immediate clinical superiority.

The superior discriminative performance of the combined model (AUC = 0.904 in external validation) compared with the individual clinical or radiomics models suggests that integrating quantitative imaging features with conventional clinical variables may improve early risk stratification in AIS. Nevertheless, the current findings require cautious interpretation and further validation before this approach can be considered broadly generalizable or ready to replace existing clinical assessment strategies.

The clinical utility of our predictive model extends beyond risk stratification to inform targeted therapeutic interventions. Future research directions should prioritize the development of personalized treatment algorithms guided by model predictions, including targeted anti-inflammatory interventions to mitigate neuroinflammatory cascades, combined with intensive glycemic control strategies to minimize metabolic disturbances and their deleterious effects on ischemic brain tissue. Emerging innovations in neuroprotective pharmacotherapy and regenerative medicine approaches may provide novel therapeutic avenues for AIS management, particularly when guided by predictive models that identify patients most likely to benefit from specific interventions. Additionally, comprehensive long-term follow-up studies incorporating serial imaging and functional assessments could identify additional prognostic factors influencing stroke progression, enabling healthcare teams to develop increasingly precise, individualized treatment protocols and optimize rehabilitation strategies for improved functional outcomes.

## Limitations and future directions

### Study limitations

Several limitations should be acknowledged in this study. First, this was a retrospective study with a relatively limited sample size. The final combined model included 4 predictors with 22 progression events in the training cohort, yielding an events-per-variable ratio of 5.5, which raises concern about model instability and overfitting despite bootstrap validation. Second, significant baseline differences were observed between the training and external validation cohorts, indicating inter-center heterogeneity. In addition, the different enrollment periods between centers may have introduced temporal bias related to evolving AIS management. Furthermore, only inter-observer reproducibility was evaluated, whereas intra-observer reproducibility was not separately assessed. Third, the study focused on 30-day progression rather than longer-term functional outcomes such as the 90-day modified Rankin Scale; therefore, the broader prognostic value of the model remains to be determined. Fourth, the radiomics analysis was based on a single MRI sequence (DWI), and the preprocessing details available from the retrospective workflow were limited, including the absence of prospectively standardized resampling and intensity normalization parameters. Future studies incorporating multimodal MRI and more standardized preprocessing procedures may further improve reproducibility and predictive performance.

### Future research directions

Future research should prioritize large-scale, multicenter prospective validation studies to establish clinical utility and guide implementation into routine practice. Additionally, extending the model to incorporate multimodal imaging data, predict treatment response to specific interventions, and develop dynamic prediction capabilities that update based on clinical course would significantly enhance its clinical value. Investigation of the biological basis underlying radiomics features and implementation science studies focusing on optimal integration into clinical workflows represent important research directions for translating this predictive tool into improved patient outcomes.

## Conclusion

This study developed a radiomics-clinical nomogram for predicting 30-day progression risk in patients with AIS. By combining quantitative imaging features with clinical predictors, the model showed promising predictive performance in both the training and external validation cohorts. The web-based deployment facilitates immediate clinical translation, offering a practical tool for personalized stroke management. These findings support the integration of radiomics into routine stroke care and provide a foundation for precision medicine approaches in acute stroke management.

## Data Availability

The raw data supporting the conclusions of this article will be made available by the authors, without undue reservation.

## Glossary

NIHSS: National Institute of Health Stroke Scale
LAA: Large-artery atherosclerosis
EH: Essential Hypertension
T2DM: Type 2 Diabetes Mellitus
APOB: Apolipoprotein B LP(a), Lipoprotein (a)
HCY: Homocysteine
LDL-C: Low-Density Lipoprotein Cholesterol
HDL-C: High-Density Lipoprotein Cholesterol
EO: Eosinophils
BA: Basophils
RBC: Red Blood Cells
HGB: Hemoglobin
MPV: Mean Platelet Volume
PLCR: Platelet-large Cell Ratio
Lp-PLA2: Lipoprotein-associated Phospholipase A2
NE: Neutrophils
WBC: White Blood Cells
MO: monocytes
PLT: Platelets
TC: Total Cholesterol
TG: Triglycerides
LY: Lymphocytes
PCT: Plateletcrit
IQR: Interquartile ranges
