# Supplementary material for: A web-based radiomics nomogram combining MRI features and clinical data for predicting 30-day progression of acute ischemic stroke: a two-center study
Source: Front Neurol. 2026 Aug 10;17:1774830. doi: 10.3389/fneur.2026.1774830 (PMC13500313; doi:10.3389/fneur.2026.1774830)
Supplement: Supplementary file 1 [file Table_1.docx]

**Supplementary Table S1.** Comparison of baseline characteristics between the training cohort and the external validation cohort.

| Variables | Total｜(N=254) | Training cohort｜(N=103) | External validation cohort｜(N=151) | χ2/Z/t | P Value |
| --- | --- | --- | --- | --- | --- |
| Age(Yrs) | 68.40 ± 11.49 | 63.36 ± 9.04 | 71.83 ± 11.73 | 6.185 | <0.001 |
| Gender |  |  |  | 0.330 | 0.566 |
| - Male | 168 (66.14%) | 66 (64.08%) | 102 (67.55%) |  |  |
| - Female | 86 (33.86%) | 37 (35.92%) | 49 (32.45%) |  |  |
| LAA |  |  |  | 25.220 | <0.001 |
| - NO | 56 (22.05%) | 39 (37.86%) | 17 (11.26%) |  |  |
| - YES | 198 (77.95%) | 64 (62.14%) | 134 (88.74%) |  |  |
| NIHSS | 4.00 [3.00;6.00] | 4.00 [3.00;7.00] | 3.00 [2.00;6.00] | -4.127 | <0.001 |
| EH |  |  |  | 0.870 | 0.351 |
| - NO | 115 (45.28%) | 43 (41.75%) | 72 (47.68%) |  |  |
| - YES | 139 (54.72%) | 60 (58.25%) | 79 (52.32%) |  |  |
| T2DM |  |  |  | 2.712 | 0.100 |
| - NO | 170 (66.93%) | 75 (72.82%) | 95 (62.91%) |  |  |
| - YES | 84 (33.07%) | 28 (27.18%) | 56 (37.09%) |  |  |
| HCY（umol/L） | 12.50 [9.60;16.46] | 13.55 [10.79;16.75] | 11.50 [8.34;16.20] | -2.841 | 0.005 |
| LDL-C（mmol/L） | 2.65 ± 0.95 | 2.85 ± 1.03 | 2.51 ± 0.86 | -2.868 | 0.004 |
| HDL-C（mmol/L） | 1.23 [1.03;1.51] | 1.08 [0.92;1.25] | 1.42 [1.15;1.71] | 7.431 | 0.000 |
| TG（mmol/L） | 1.38 [0.93;1.87] | 1.40 [1.05;1.78] | 1.34 [0.83;2.03] | -0.702 | 0.483 |
| APOB（g/L） | 0.92 ± 0.26 | 0.91 ± 0.27 | 0.92 ± 0.25 | 0.379 | 0.705 |
| TC（mmol/L） | 4.56 [3.84;5.33] | 4.55 [3.78;5.30] | 4.56 [3.92;5.37] | 0.116 | 0.909 |
| WBC（10^9/L） | 6.69 [5.60;7.80] | 6.30 [5.50;7.50] | 6.95 [5.82;8.06] | 2.328 | 0.020 |
| NE（10^9/L） | 4.38 [3.49;5.64] | 4.20 [3.49;5.12] | 4.46 [3.50;5.77] | 1.430 | 0.153 |
| LY（10^9/L） | 1.50 [1.17;1.89] | 1.50 [1.30;1.80] | 1.50 [1.10;1.92] | -0.572 | 0.568 |
| MO（10^9/L） | 0.39 [0.30;0.46] | 0.34 [0.27;0.43] | 0.41 [0.31;0.49] | 3.160 | 0.002 |
| EO（10^9/L） | 0.10 [0.05;0.18] | 0.09 [0.05;0.15] | 0.10 [0.05;0.20] | 0.971 | 0.332 |
| BA（10^9/L） | 0.02 [0.01;0.04] | 0.01 [0.00;0.02] | 0.03 [0.02;0.04] | 9.293 | <0.001 |
| RBC（10^12/L） | 4.47 [4.04;4.78] | 4.59 [4.25;4.86] | 4.29 [3.92;4.72] | -3.204 | 0.001 |
| HGB(g/L) | 136.00 [125.00;148.00] | 140.00 [130.50;149.00] | 133.00 [121.00;147.00] | -2.879 | 0.004 |
| PLT（10^9/L） | 206.00 [166.00;240.00] | 206.00 [176.00;237.50] | 206.00 [154.00;245.50] | -0.359 | 0.720 |
| lp-pla2（ng/ml） | 226.00 [130.00;435.00] | 212.00 [117.50;419.00] | 232.00 [134.00;443.00] | 1.526 | 0.127 |
| PCT(%) | 0.22 ± 0.06 | 0.22 ± 0.06 | 0.21 ± 0.06 | -0.674 | 0.501 |
| MPV(fl) | 10.33 ± 1.17 | 10.60 ± 1.44 | 10.15 ± 0.90 | -3.048 | 0.003 |
| PLCR | 26.65 [21.20;32.80] | 29.20 [23.55;35.65] | 25.00 [20.00;30.40] | -4.077 | <0.001 |
