# Supplementary material for: A web-based radiomics nomogram combining MRI features and clinical data for predicting 30-day progression of acute ischemic stroke: a two-center study
Source: Front Neurol. 2026 Aug 10;17:1774830. doi: 10.3389/fneur.2026.1774830 (PMC13500313; doi:10.3389/fneur.2026.1774830)
Supplement: Supplementary file 2 [file Table_2.docx]

**Supplementary Table S2.** Radiomics features

| Feature_ID | MaZda_Feature_Name | Texture_Category | Coefficient | Direction of Association with PIS |
| --- | --- | --- | --- | --- |
| Feature 1 | _Area_S(0,1) | GLSZM | 0.065 | Positive |
| Feature 2 | S(1,-1)SumOfSqs | GLCM | -0.253 | Negative |
| Feature 3 | _Area_S(0,2) | GLSZM | 0.146 | Positive |
| Feature 4 | S(3,-3)Correlat | GLCM | 0.341 | Positive |
| Feature 5 | S(3,-3)InvDfMom | GLCM | 0.085 | Positive |
| Feature 6 | S(0,4)Correlat | GLCM | 0.055 | Positive |
| Feature 7 | Horzl_LngREmph | GLRLM | 0.029 | Positive |
| Feature 8 | WavEnLH_s-4 | Wavelet | -0.067 | Negative |
